# A Literature Review of Concrete Ability to Sustain Strength after Fire Exposure Based on the Heat Accumulation Factor

**DOI:** 10.3390/ma14164719

**Published:** 2021-08-21

**Authors:** Michał Pasztetnik, Roman Wróblewski

**Affiliations:** Faculty of Civil Engineering, Wroclaw University of Science and Technology, 50-370 Wrocław, Poland; michal.pasztetnik@pwr.edu.pl

**Keywords:** concrete, compressive strength, high temperature, fire temperature, residual strength, heat accumulation factor

## Abstract

Concrete is susceptible to damage during and after high-temperature exposure (most frequently in fire). The concrete partial strength re-gain after a high-temperature exposure obtained by the rehydration process is undoubtedly an advantage of this construction material. However, to use fire-damaged concrete, one has to know why the strength deteriorates and what makes the partial re-gain. Within this framework, the paper aims to find what factors influence the strength re-gain. Moreover, an attempt is made to introduce a measure collecting various influences such as the modified heat accumulation factor—accounting only for that which is important for the process, the temperature decomposing cement paste (i.e., above 400 °C). Several factors, i.e., peak temperature, heating time and rate, cooling regime, post-fire re-curing, concrete composition, age of concrete at exposure, porosity, load level at exposure, and heat accumulation are presented by their influence on the relative residual compressive strength, i.e., a portion of initial strength that is obtained after temperature exposure and strength re-gain. Since the relative strength unifies various concretes, a more general assessment and discussion are presented based on the experimental results and correlation factors. As fundamental influences determining the residual strength, the heating time, peak temperature, cooling, or post-heating re-curing regimes are found with the load level at exposure being inadequately examined. This paper also shows the superiority of the modified heat accumulation factor, but the results obtained are not satisfactory, and additional experimental data are necessary to develop a theoretical model of the residual strength.

## 1. Introduction

Concrete provides the best fire resistance out of typically used construction materials. Compared to timber or steel, concrete has low thermal conductivity, high heat capacity, and its strength degrades slower with increasing temperature. As a result, it is a material that performs well not only as a separator between fire-affected spaces but also as a material for elements that need to perform under extreme thermal conditions. The vast majority of concrete structures can withstand fire conditions for the designed duration. This is due to the fact that concrete properties at high temperatures are well known and examined [1].

Standards provide safe approaches and mathematical models [2,3,4] that help design concrete structures for fire safety. Stress–strain curves for concrete exposed to high temperatures are also provided [5,6,7], and the behavior of concrete structures, elements, and sections are widely investigated, e.g., [8,9,10]. The research and design effort result in structures that handle fires so well that the question arises: Can this structure be used as it was before the fire?

Destructive and non-destructive assessment of strength is possible [11,12,13,14], but providing enough information for structural calculations is a crucial issue. The temperature field inside the concrete element depends on many uncertain parameters, and this results in residual properties that are difficult to estimate. Therefore, to answer any question about residual properties, theoretical models would be helpful.

From both research and practical perspectives, the residual strength of concrete is a complex matter [15], because many factors must be considered, e.g., peak temperature, heating time, rate of increase in temperature, concrete composition, and others).

Since concrete is a composite material (composed of aggregate, cement, water, and additives), its final properties vary depending on the ratio and type of components used, but subjected to fire, other variables influence the properties. When a cement paste is exposed to high temperatures, the following physicochemical reactions are observed [16]:

- At 100 °C, bound water is being evaporated;
- At 180 °C, hydrate calcium silicate is beginning to dehydrate;
- At 500 °C, the decomposition of calcium hydroxide is observed;
- At 700 °C, the decomposition of calcium silicate hydrate occurs.

These phenomena cause a degradation of the mechanical properties of the cement paste [17]. In addition, the thermal expansion of the aggregate leads to an increase in internal stresses and can result in microcracks of the concrete body. The deterioration of mechanical properties can be represented by the relative residual strength. Compressive and tensile strength degrade rapidly as they halve at 400 °C and 300 °C, respectively [18]. The decline is not only a function of the peak temperature but also of the heating time and rate. It was proven that a higher heating rate results in lower relative strength at the same peak temperature [19]. Heating time is also crucial, as maintaining a low temperature for a long time can cause more damage to the concrete than a higher peak temperature for a short time. What should be remembered is the fact that concrete becomes less brittle after exposure to a high temperature (intrinsic length increases) [20]. The size effect can influence the tensile strength. Although it has a marginal effect on compression [21], it makes the tensile test results sample size dependent. It was found that the strain rate does not influence compressive behavior [22].

After the concrete is cooled down to ambient temperature, it retains residual strength that is affected not only by the mentioned factors but also by the cooling regime, post-fire re-curing regime, and time. There exists a correlation between strength at high temperature and residual strength, but they cannot be treated in the same way.

This review paper summarizes up-to-date progress in experimental research on the residual strength of concrete after high-temperature exposure. The results of the tests on the most important factors that influence concrete residual strength are presented and discussed in the following sections of the article. Each section is dedicated to a separate factor. Although there are consistent results for obvious factors, such as peak temperature and heating time, the influence of less pronounced factors, such as the w/c ratio and cement type, still requires further research. Furthermore, current research lacks a general approach to the influences that will lead to a function or algorithm capable of assessing the residual strength of concrete.

## 2. Peak Temperature

According to [2], the strength of the concrete at high temperature is a function of only the temperature that the concrete reaches. With residual strength, more factors need to be considered, but peak temperature is crucial amongst them. Extensive research was done to connect the peak temperature and residual strength of the concrete (Table 1). Comparison between the results is very limited, as factors besides peak temperature are often disparate for different authors.

**Figure 1:**
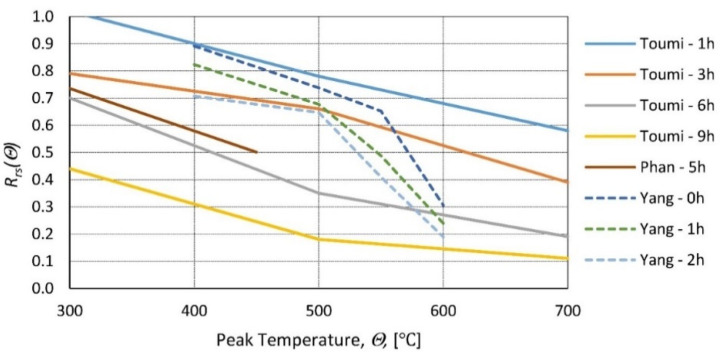
Relative residual strength of concrete as a function of peak temperature for the different times maintained at peak temperature of 1, 3, 5, 6, and 9 h according to [23]—Toumi, [25]—Phan, and 0, 1, and 2 h according to [24]—Yang.

In [23,24,25], cylindrical concrete samples were heated to temperatures ranging from 100 to 450 °C; then, after reaching steady state, they were cooled to room temperature inside the furnace, and the compressive strength was tested (Figure 1). In [26], research was performed for a very broad range of temperatures (200, 400, 600, 800, and 1000 °C). Concrete samples of size 40 mm × 40 mm × 160 mm at the age of 28 days were placed in the furnace and kept at the target temperature for 1.5 h; then, they were taken out and left to cool in an ambient environment. Compressive tests were performed on prism halves resulting from flexural tests.

**Figure 2:**
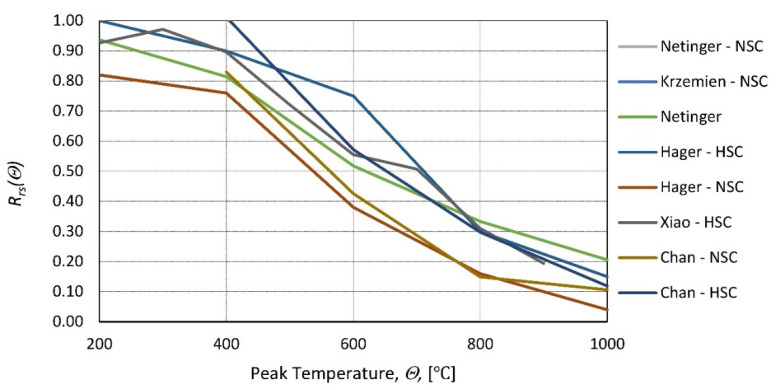
Relative residual strength of different types of concrete (NSC and HSC) as a function of the peak temperature according to [26]—Netinger, [27]—Hager, [28]—Krzemien, [29]—Xiao, and [30]—Chan.

**Figure 3:**
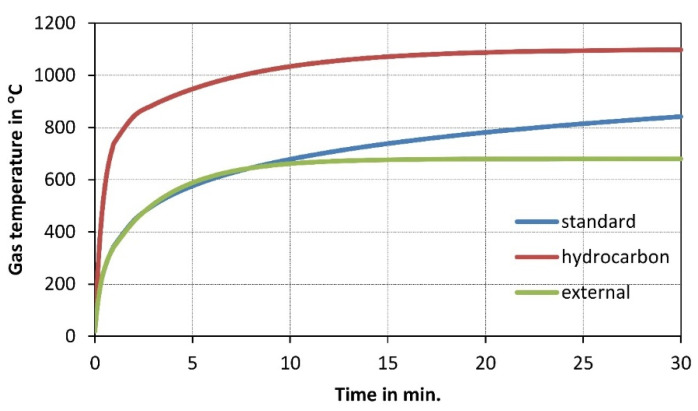
Development of gas temperature in hydrocarbon, external, and standard fires according to [35].

The HSC (high-strength concrete) samples [27] and the NSC (normal-strength concrete) samples [28] were heated in a furnace test (to temperatures: 200, 400, 600, 800, and 1000 °C). A low heating rate of 0.5 °C/min was applied, and the peak temperature was maintained for 3 h. Subsequently, the samples were cooled inside the furnace, taken out, and tested (Figure 2). In [29], the samples were tested at 10 set temperature values (20, 100,…, 900 °C). The temperature was increased according to the standard fire curve (Figure 3), and the peak temperature was maintained for 3 h. Then, the furnace door was opened, and the samples were cooled inside before compressive tests (Figure 2). In [30], HSC and NSC samples were heated to temperatures ranging from 400 to 1200 °C. After the specimens were allowed to cool naturally to room temperature, compressive tests were performed (Figure 2). In [31], the residual strength of NSC and HSC after exposure to elevated temperatures (from 200 to 600 °C) was compared.

**Figure 4:**
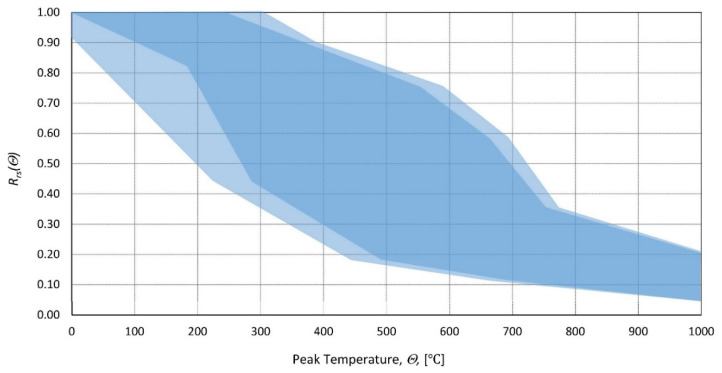
Range of the relative residual strength of concrete as a function of peak temperature. Collection of data from different heating rates, types of concrete, sample sizes, etc. presented by various authors.

Moreover, the results demonstrate that the relative loss of strength is higher for HSC than for NSC. A similar conclusion was reached in [32,33]. Correlation factors were calculated for all the data collected, with respect to the heating time at peak temperature (Table 2). The Pearson and Spearman factors signify a strong negative linear relationship between relative residual strength and peak temperature. The Kendal coefficient supports this observation, as it indicates a monotonic relationship. The graphic representation of the collected results is presented in Figure 4. Residual strength behaves in a way similar to changes in concrete strength at high temperature according to, e.g., [2]. Varying the test conditions and concrete composition in the research considered in Figure 4 can make a substantial difference in residual strength for peak temperatures ranging from 300 to 750 °C. For peak temperatures lower than 300 °C, almost no loss is observed, and for peak temperatures higher than 750 °C, residual strength is almost equal to strength at high temperatures, meaning that concrete damaged to a very high degree exhibits a smaller ability to regain strength. Taking into account all this, the residual strength function for temperatures up to 300 °C and more than 750 °C can be derived based on the peak temperature only, but for the remaining range, a more accurate function would require considering other factors, which were mentioned further in this paper. In [34], a function was proposed to relate the residual strength with the maximum temperature, but it is considered as a rough approximation.

## 3. Heating Time

**Figure 5:**
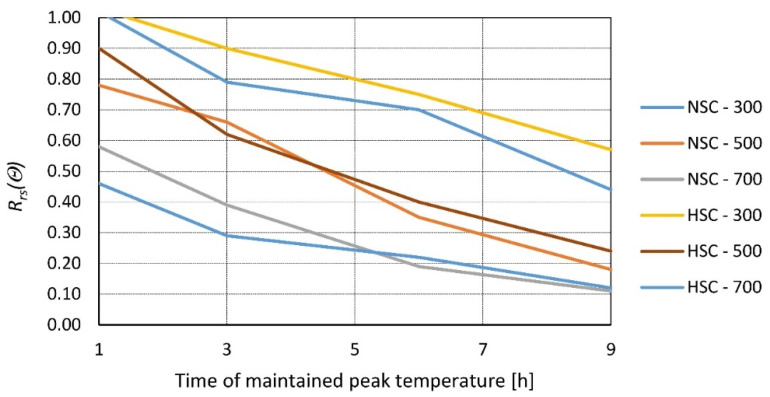
Relative residual strength of NSC and HSC as a function of time maintained at the peak temperature of *θ* = 300, 500, and 700 °C according to [23].

The heating time, that is, how long the peak temperature is kept, is crucial in assessing the residual strength of concrete, which is demonstrated in the research presented (Table 3). In [23], this relationship is presented based on concrete made of crushed limestone and CEM I 42.5 in two variants (Figure 5):

- Normal-strength concrete (NSC) with a w/c ratio of 0.5;
- High-strength concrete (HSC) with a w/c ratio of 0.37.

**Figure 6:**
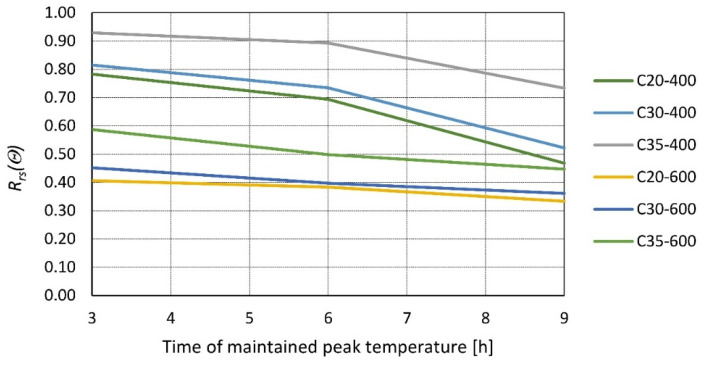
Relative residual strength of the concrete class C20/25, C30/37, and C35/45 as a function of time maintained at the peak temperature of *θ* = 400 and 600 °C according to [36].

At 28 days, samples were heated at a rate of 10 °C/min to target temperatures 300, 500, and 700 °C and kept for 1, 3, 6, and 9 h. The samples were tested after 24 h of cooling at room temperature (Figure 5). In [36], concrete samples of compressive strength of 20, 30, and 35 MPa were investigated 28 days after casting; cubic (150 mm) samples were heated to 400 and 600 °C for a duration of 3, 6, or 9 h and tested after cooling to room temperature (Figure 6).

**Figure 7:**
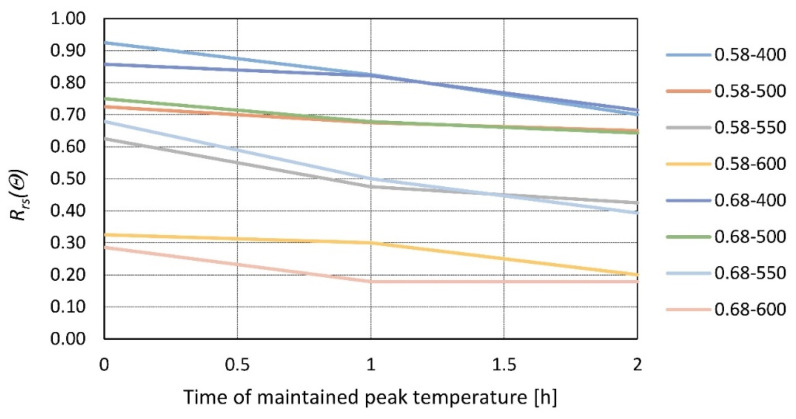
Relative residual strength of concrete with different water to cement ratios (0.58 and 0.68) as a function of time maintained at peak temperature of *θ* = 400, 500, 550, and 600 °C according to [24].

Cylindrical samples (100 mm in diameter and 200 mm high) were tested in [24] at the age of 90 days. Two water–cement (w/c) ratios were used: 0.58 and 0.68. The heating rate was set at 2.5 °C/min to achieve peak temperatures of 400, 500, 550, and 600 °C. Temperatures were maintained for 0, 1, and 2 h, and then strength tests were performed after 7 days of cooling (Figure 7).

Cylindrical specimens were also tested in [37] by exposing them to temperatures ranging from 200 to 600 °C. The heating rate was set at 5 °C/min, and the exposure time varied from 1 to 6 h. Compressive tests were performed directly after cooling down. Another test is reported in [38]. Cubic samples (100 mm) were heated to temperatures from 200 to 800 °C with various heating rates and exposure times. The tests were performed after 14 days of re-curing. It was found that the main strength loss occurs within the first two hours of high-temperature exposure, and later, the impact is minimal. Comparing all of the data, it is clear and confirmed that a longer heating time deteriorates the residual strength of concrete, where most of the loss occurs in the first two hours. The correlation between residual strength and time maintained at peak temperature was calculated for 500 °C (as it provides the broadest range of results), and the factors are presented in Table 4. A strong, negative, monotonic, and linear relationship is evident.

**Figure 8:**
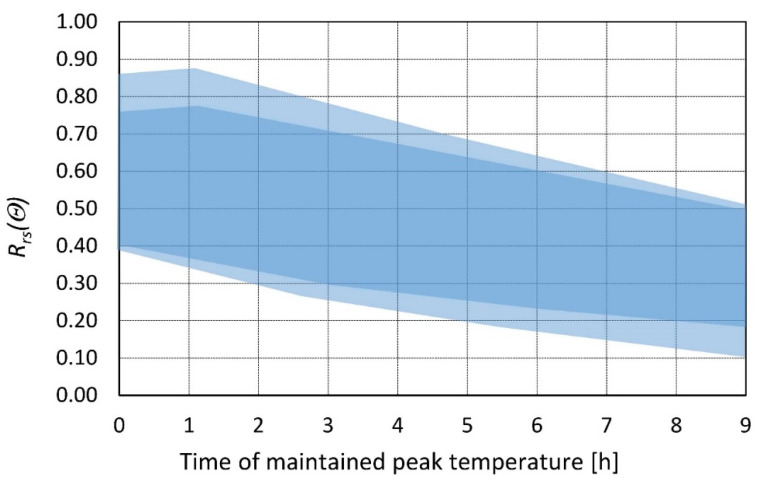
Range of the relative residual strength of the concrete as a function of time maintained at peak temperature. Collection of data from different heating rates, types of concrete, sample sizes, etc. presented by various authors.

Data reported by different authors result in the strength dispersion presented in Figure 8. Peak temperature and other variables impact the residual strength, so the data range is very wide. The bottom line represents the loss of residual strength for higher temperature ranges (700 °C) and the top line represents the loss of residual strength for lower peak temperatures (300 °C). This suggests that it is impossible to develop a proper function based only on time maintained in peak temperature. However, the derivative of this function is constant in segments and does not depend on the peak temperature value. The first segment is from 0 to 2 h (rapid loss of strength) and from 2 h onward (minimal loss of strength). This derivative gives a chance to isolate the influence of heating time on residual strength in the form of a coefficient implemented on an already known strength value with longer (or shorter) heating time.

## 4. Heating Rate

Although the gas temperature in a fire can rise extremely fast, as presented in Figure 3 [35], the temperature inside an element does not follow the same rate. The size of the concrete element, the high specific heat, and the thermal conductivity of around 1 W/mK result in very slow heat transfer throughout the element [2]. Additionally, the specific heat doubles at around 100 °C because the water changes state and the thermal conductivity decreases with rising temperature. Thus, only the external part of the cross-section is exposed to very high temperature, while internal parts record noticeably lower ones.

Although there are very different heating rates (3.5 and 10 °C/min) in [39], the test results obtained suggest a minimal influence of the heating rate. In [38], the influence of the heating rate was confirmed. However, no clear trend could be drawn in peak temperatures up to 600 °C. Beyond that level of peak temperature, no substantial influence was observed for different heating rates. The 2.5 °C/min rate applied in [24] results in a sharper decline in strength than with the 10 °C/min proposed in [23]. This leads to the conclusion that the damage caused by the high heating rate makes concrete less prone to further deterioration, while the low rate results in concrete that is susceptible to the effects of prolonged high temperature exposure. However, an immediate relationship has not been proven. Correlation factors between the heating rate and residual strength were calculated for the collected data, and the factors are presented in Table 5. Low positive values suggest a minimal influence in favor of the higher heating rate.

## 5. Cooling Regime

After heating or a fire, an element subjected to high temperature cools down, and how it happens is called the cooling regime. There exist three basic types of the regime used in tests:

- High-temperature environment cooling—concrete and environment maintain a high temperature for a long time, and slow temperature lowering from peak value to ambient is performed. This type of cooling corresponds to what happens in the inner parts of the fire-affected elements. The relatively high thermal capacity of concrete and low thermal conductivity cause temperature changes inside elements to be slow, both during heating up and cooling down.
- Cooling at the ambient temperature environment—hot concrete is kept at room temperature for cooling. This can be equated to the occurrence in the outer parts of fire-affected elements.
- Water cooling—hot concrete is treated with cold water and cooled down. This type can be compared with the outer parts of fire-affected elements covered by water used to extinguish the fire.

**Figure 9:**
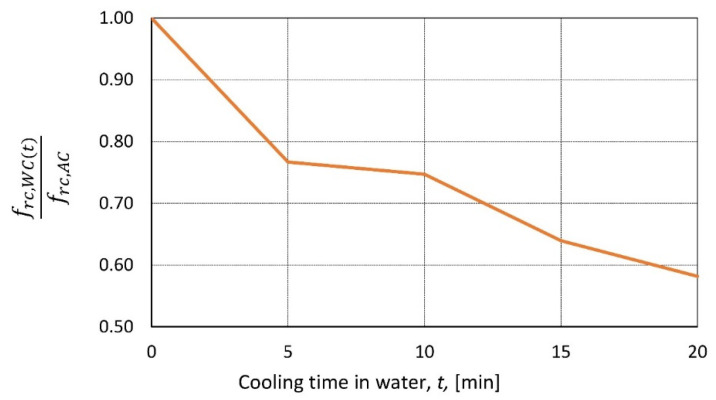
$\frac{f_{rc,WC\;}\left(t\right)\;}{f_{rc,AC\;}\;}$ as a function of the cooling time in water according to [40].

According to the available research (Table 6), the degradation of the mechanical properties depends on the type of cooling. Ambient temperature and water cooling were used in [40] but with cylindrical samples (diameter/height = 100/200 mm) heated to 330, 450, and 550 °C and removed from the furnace. Then, five types of cooling were performed: ambient temperature air cooling, water cooling by immersing samples in 15 °C water for 5, 10, 15, and 20 min and then air-cooled to room temperature. The next day, strength tests were performed. When air-cooled samples were compared with top-water-cooled samples, it appeared that peak temperature was not important for the decline rate of relative residual strength. Strength loss depends mainly on the duration of immersion in water (Figure 9).

Similar tests are presented in [41,42,43], and the conclusions reached are the same. In [44], the impact of rapid water cooling on concrete made with the addition of slag was tested. Specimens were exposed to elevated temperatures of 400 and 800 °C and cooled in air or 20 °C water. For 400 °C, the strength loss of all water-cooled samples reaches an additional 20% compared to air cooling. It is interesting to note that for 800 °C samples made with OPC (ordinary Portland cement), the loss of an additional 14% points is observed when water-cooled samples made with a partial replacement of OPC by slag lose only 4–5% points. This phenomenon can be explained by the rehydration of CaO in Ca(OH)_2_ accompanied by the expansion and thus further deterioration of concrete.

In [45], cubic samples (100 mm) were tested by exposing them to elevated temperatures (from 50 to 700 °C) and then cooling in two ways: by leaving them to cool in the furnace and by immersing them in water. The conclusion was drawn that air cooling results in higher residual strength, especially in the 400–500 °C peak temperature range.

Cubic specimens (100 mm) specimens were tested 90 days after casting in [46]. Temperature exposure was carried out in an electric furnace with the heating rate set at 10 °C/min for the first 100 °C and 20 °C/min from 100 to 800 °C. After reachifng peak temperature, samples were divided. One part was taken out of the furnace and cooled at room temperature; the second part was left in the turned-off furnace (door closed). Then, the tests were performed in two groups: directly after and 30 days after cooling. The results provide interesting facts: high-temperature cooling causes an additional dehydration of hardened cement and further deterioration of the strength (Table 7). Air cooling stopped the dehydration process but caused more internal cracks due to the temperature gradient. When comparing results obtained directly after cooling, dehydration had a greater impact than internal cracks but is also more reversible. After 30 days of re-curing, cement rehydrates and concrete regains most of its initial strength, while internal cracks caused by temperature gradient are unable to close.

In [47], water and furnace (air) cooling is compared by heating the NSC and HSC samples to 800 °C at a rate of 5–7 °C/min and cooling them in either a turned-off furnace or in a water tank. The difference between the cooling methods was visible for the residual strength tested directly after cooling. The NSC strength was reduced to 0.32 for water and 0.45 for air cooling. The impact of the cooling regime on the residual strength of HSC was less pronounced, as the strength was reduced to 0.21 for water and 0.26 for air cooling.

A difference in the cooling regime for various peak temperatures is presented in [48]. Temperatures ranged from 200 to 800 °C. The residual strength resulting from slow air cooling was higher compared to water cooling for all temperature cases by 4–7% points. The difference was not substantial but noticeable. In [49], the influence of water cooling on the residual strength of concrete was tested. The samples were cooled in two ways: air-cooled in a furnace and water-cooled by sprinkling water for 30 min directly after temperature exposure. Compressive tests were performed one month after high-temperature exposure. The influence of the cooling regime was found for exposure temperature from 200 to 600 °C; water cooling resulted in lower residual strength by 5–7%. Only for 800 °C was there a difference of 1.5% (air cooling resulted in lower residual strength). The same cooling regime was used in [50]; later samples were tested after different re-curing times (0, 30, 60, and 90 days). The results proved that water cooling lowers the residual strength tested directly after cooling (especially after exposure to temperatures higher than 600 °C), but after re-curing for a longer period, the difference caused by the cooling method was minimal (Table 8).

Three cooling methods were examined in [51]: that is, furnace air cooling, room temperature air cooling, and full immersion water cooling after exposure to 700 °C. Compressive tests were performed after the samples reached ambient temperatures. In contrast to the other experiments, samples cooled in water demonstrated the highest residual strength, whereas furnace and room temperature cooling showed very similar, but lower, results.

The cooling regime is an important factor in the evaluation of the residual strength of the concrete. According to [52], rapid cooling produces more internal damage due to the temperature gradient [53,54], but it stops dehydration processes. The slower cooling process results in longer exposure to high temperatures and longer dehydration. It is worth mentioning that water cooling results in lower weight loss [55].

The available data indicate that water cooling results in lower residual strength directly after cooling. Nevertheless, after post-fire re-curing, the difference between residual strengths for different cooling types becomes small and can be neglected.

## 6. Post-Fire Re-Curing Effect on Residual Strength

The recovery of the strength of concrete due to post-fire re-curing is important when assessing the residual strength of concrete, and it was proven in many articles (Table 9). This restoration can be attributed to the rehydration of cement that was dehydrated at high temperatures [56]. An essential factor in this phenomenon is moisture, which is similar to the initial curing of concrete. While full recovery is impossible (only the pore structure can return to pre-fire state), mechanical properties can return to surprisingly high levels. Some of the concrete phases form active products at elevated temperatures, such as limes and calcium silicates; the effect of water and carbon dioxide on these can contribute to increased residual properties [57]. Post-fire re-curing methods can be classified into three basic categories: water post-fire re-curing, air–water post-fire re-curing, and air post-fire re-curing. [58].

The difference between them is defined by the supply of water, from full immersion for a whole amount of time to no water at all. In [59], concrete samples were exposed to various temperatures ranging from 200 to 800 °C and then re-cured in air. Compressive tests were performed after 1, 7, 30, and 90 days after cooling. The results presented for 200 and 400 °C show that there is a rapid decrease in residual strength for the first week and then a slow increase. At 90 days, re-cured residual strength is higher than the initial residual strength.

**Figure 10:**
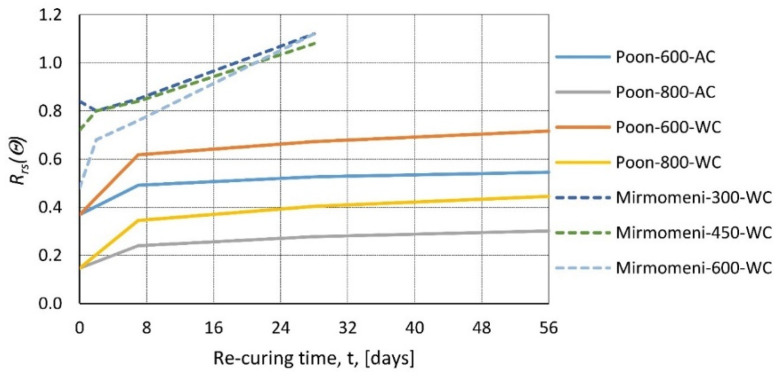
Relative residual strength of concrete [60] and self-compacting concrete [64] as a function of re-curing time (t) for different re-curing methods (AC—air re-curing, WC—water re-curing) and different peak temperatures *θ* = 300, 450, 600, and 800 °C according to [60]—Poon and [63]—Mirmomeni.

In [60], various concrete mixes were tested by heating samples to temperatures of 600 and 800 °C with a heating rate of 2.5 °C/min and a time maintained at the peak temperature of 1 h. After exposure, the specimens were tested at four different times: directly after cooling, 7, 28, and 56 days of re-curing. Moreover, two re-curing regimes were used; the first was water re-curing, where samples were cooled down to room temperature naturally and then placed in water, while the second consisted of cooling down to room temperature, soaking in water for 2 h, and air curing for the remaining time. Results show that post-fire re-curing can produce tremendous effect (Figure 10). Samples that were heated to 800 °C and water re-cured increased their compressive strength three times. The regaining of mechanical properties is rapid in the first 7 days when the average gain is 75% (compared to the test directly after cooling); later, growth is linear. At 28 days, the average increase is 100%, and at 56 days, it is 115%.

**Figure 11:**
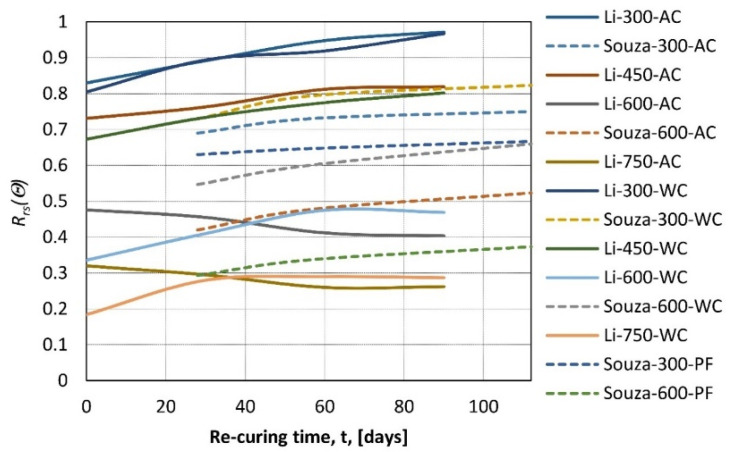
Relative residual strength of concrete as a function of re-curing time (t) for different peak temperatures *θ* = 300, 450, 600, and 750 °C, and different re-curing methods (AC—air re-curing, WC—water re-curing, PF—re-curing in a plastic film) according to [61]—Souza and [50]—Li.

The re-curing method shows differences in the first 7 days, water re-curing rates at 100% of increase, while air curing rates at 50%, but later, the growth becomes linear and very similar for both methods. In [61], concrete samples were heated up to 300 or 600 °C (heating rate 1 °C/min) and then re-cured for 28, 56, or 112 days at three different re-curing regimes. First, samples were covered in plastic film (PF) to prevent any moisture from reaching the re-curing concrete. The second was standard air curing (AC), and the third was water re-curing (WC). Then, the results were compared with the strength before heating. It was found that residual strength growth is more pronounced at the beginning stage of re-curing, and it slows over time (Figure 11). The rate at which strength is regained varies, depending on the re-curing method, with the rule that more moisture gives better results. In [62], similar research was performed, and the statement that water re-curing gives better results than air re-curing was confirmed.

In [50], the residual compressive strength was tested as a function of re-curing time and the cooling regime, samples were re-cured after fire for 0, 30, 60, and 90 days depending on peak temperature with two different cooling regimes: air cooling and water cooling. The difference between air and water cooling is most visible for the test directly after cooling: the higher the temperature, the larger the initial difference. Then, with re-curing time increasing, the method of cooling is of small relevance (Figure 11).

Self-compacting concrete samples were examined in [63] by heating to 300, 450, and 600 °C and then tested after 0, 2, 7, and 28 days after cooling. The results prove the great potential of concrete to regain strength, in some instances even exceeding strength before high-temperature exposure (Figure 10). In [64], the recovery behavior of hybrid fiber HSC after fire exposure was tested. The samples were exposed to 200 and 400 °C. After exposure, strength tests were performed directly after, 90 days after, and 180 days after in two different re-curing conditions. Air re-curing resulted in a slight regain of compressive strength, while water re-curing essentially reinstated concrete to the initial strength. It is worth noticing that the regain occurred in the first 90 days; after that time, only a small increase was recorded.

**Figure 12:**
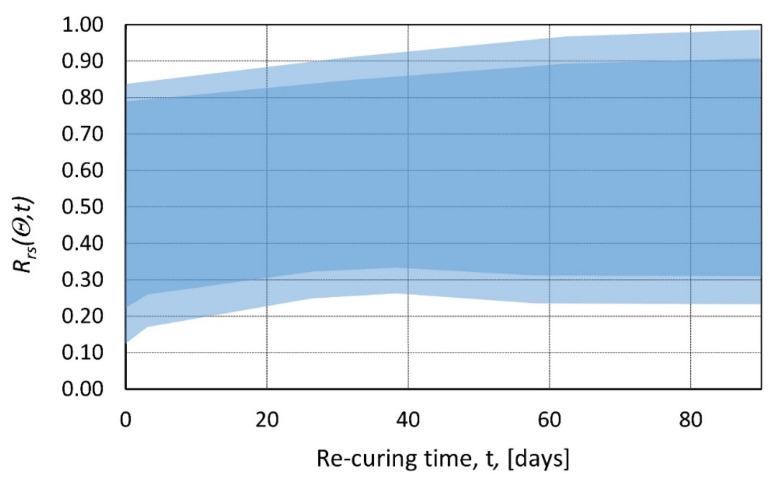
Range of the relative residual strength of concrete as a function of re-curing time. Collection of data from different heating rates, types of concrete, sample sizes, etc. presented by various authors.

Initially, the insufficient concrete strength over time can become high enough to carry the necessary stresses. Correlation calculated for re-curing time and residual strength displays a positive relation (factors presented in Table 10). The values of the factors suggest that a monotonic relationship exists but does not have to be a linear one. This fact shows that post-fire re-curing is significant and must be taken into account. It is proven that the most rapid growth of mechanical properties takes place in the first 7 days; later, it slows down similar to the logarithmic function (Figure 12). Concrete recovery coincides with the rehydration of cement, and moisture is critical. Curing methods involving water provide better results while completely sealing the moisture flow results in a much slower regain of mechanical properties [65]. Although it should be noted that in concrete heated to less than 300 °C, ongoing deterioration of concrete can be observed due to sulfate-induced expansion [66]. This phenomenon can mitigate some residual strength gain and should be taken into account.

## 7. Concrete Composition

The composition of the concrete mix determines its initial mechanical properties. The question about its influence on the residual strength was studied multiple times, as it is naturally supposed. Concrete is formed by mixing aggregates, cement, water, and additives. Furthermore, research on recycled materials was implemented, creating possibilities for future usage [67]. The influence of each component is analyzed and discussed in the following sections. As the amount of data on concrete composition is vast, a summary of the examined components in all papers analyzed in this paragraph can be found in the supplementary file attached to the paper.

### 7.1. Type of Aggregate

**Figure 13:**
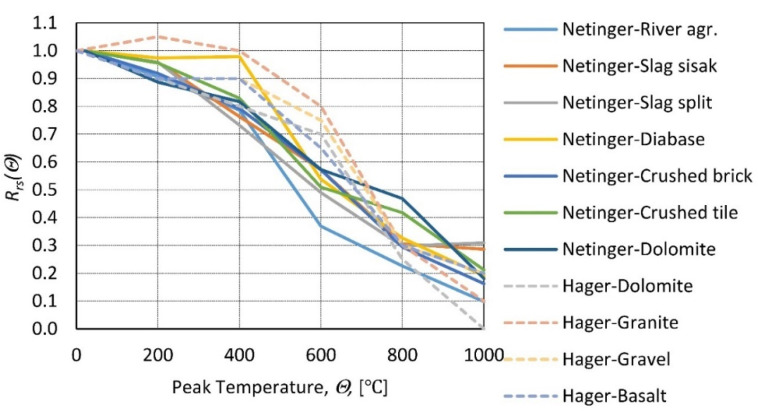
Relative residual strength of concrete as a function of peak temperature for different types of aggregate according to [27]—Hager and [26]—Netinger.

The aggregate contributes to the largest part of the concrete mix (approximately 70% in terms of volume) and is expected to have an essential influence on the behavior of concrete. A comprehensive study on aggregate behavior at elevated temperatures in [68] provides information on the phenomena that occur in concrete. An important conclusion was made that the siliceous/calcareous categorization used by [35] is not enough, as the aggregate within one of the ‘groups’ can vary significantly in terms of mechanical response to elevated temperature. The type of aggregate influence was studied in [27]: four types of HSCs were purchased and differed solely by aggregate type (Figure 13). Then, after exposure to high temperature, tests were performed to identify differences in residual strength directly after cooling. The results show that the relative residual compressive strength is very similar for all types of aggregates at a peak temperature above 600 °C; at lower temperatures, there are slight differences favoring granite [69]. Samples with granite, heated to 300 °C, show a higher residual strength than limestone. For 600 °C, this difference diminishes. Considering the gain in granite strength in the lower temperature range, the conclusion that all aggregate behaves similarly can be drawn. Furthermore, the type of aggregate does not influence the relative residual strength. These results show a very similar behavior compared to [27] for a lower temperature register (up to 300 °C). In [26], seven types of aggregate were tested, samples were heated to target temperatures and, after 1.5 h of heating, left to cool at room temperature. After the samples cooled, compressive tests were performed. Figure 13 presents a decrease in relative residual strength with respect to peak temperature following a similar trend for all aggregate types. A conclusion could be made that the aggregate type does not influence the deterioration of mechanical properties; all tested types show similar degradation over time.

**Figure 14:**
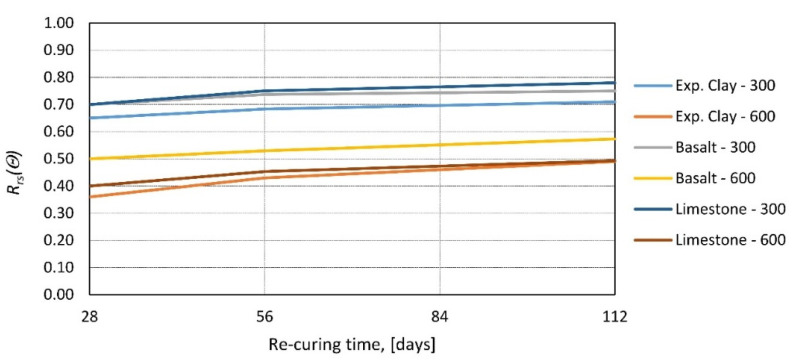
Relative residual strength of concrete as a function of re-curing time for different types of aggregate and different peak temperatures *θ* = 300 and 600 °C according to [61].

In [61], residual strength research was performed by testing concrete with three different types of aggregate: expanded clay, basalt, and limestone. Specimens were heated to 300 and 600 °C and, after cooling to room temperature, tested. The results bring the same conclusion that the type of aggregate plays a minimal role in the relative residual strength of concrete. All three types of samples had very comparable relative strengths, and regrowth follows an analogous rate (Figure 14).

In [70], a comparison was made between crushed and river aggregates. Both had similar mineralogical compositions (river with negligible higher SiO_2_ content) after exposure to elevated temperatures (from 200 to 1000 °C). The results showed that the crushed aggregate regained a higher residual strength value. In [71], research on the thermomechanical behavior of baritic concrete exposed to high temperature was conducted, and the results showed that it behaves very similar to regular concrete. In contrast to the negligible influence of the type of aggregate in normal-weight concrete, the authors of [72] researched the influence of high temperature on heavy-weight concrete properties. As a result, ilmenite concrete was found to have much higher residual strength than regular gravel concrete.

In [49], the recycled concrete aggregate was exposed to temperatures of 200 to 800 °C, and the residual strength was tested after 30 days of re-cure. Specimens differed only in aggregate type, and three types were considered: coarse, fine, and 50/50 fine and coarse. Results proved that the aggregate size did not influence the residual strength. In [73], samples made with three different aggregate types: river gravel, crushed limestone, and RCA (recycled concrete aggregate) were tested by exposing them to elevated temperatures (250, 500, 750 °C) and then, after natural cooling, their residual prosperities were tested. The results suggest that the concrete made with crushed limestone and RCA had higher relative residual strength than the river gravel. At [42], coarse RCA was also tested using different ratios (from 0 to 100%) of coarse aggregate, and the conclusion was drawn that its content is not significant for residual strength (peak temperatures of 200 to 800 °C). Similar results were reached in [74,75,76,77], although two subsequent articles pointed out that there is a small residual strength difference in favor of regular concrete. A similar experiment was carried out in [78], except for fine aggregate, which was also made from recycled concrete. The conclusion that RCA concrete has higher residual strength than normal concrete (especially for 50, 70, and 100% replacement ratios) was reached and later confirmed with a very similar test in [79]. However, in [80], contrary results were reached: for every 1% of RCA replacement, the residual strength was reduced by 0.2%. This discrepancy can be accounted for by different RCA origins, and it is of importance in residual behavior.

In [81], tests were performed on concrete made with coarse aggregate made from recycled ceramic exposed to elevated temperatures (200, 400, 600 °C), and the researchers concluded that specimens with replacement with RCCA (coarse aggregate made from recycled ceramic) had improved relative residual strength. Crushed brick aggregate was tested in [82] by replacing 30% of standard aggregate in concrete mix and exposing it to elevated temperatures. The result proved that concrete made in this way behaves very similarly to the control mix. The possibilities of replacing fine aggregate with non-ground granulated blast-furnace slag and coal bottom ash were checked [83]. Samples were made with different replacement ratios (ranging from 10 to 50%) and exposed to a temperature of 800 °C. The results showed that there are no significant differences in residual strength for different types and ratios of aggregate replacement. In [84], siliceous and calcareous aggregates were used to study the influence on the residual strength of concrete. A suggestion was made that the type of aggregate was an important factor of residual strength and that siliceous/calcareous division was not sufficient to receive precisely characterized concrete behavior.

Research carried out on the influence of aggregate type on relative residual strength proves that the limited influence exists and the change is especially noticeable for heavy-weight concrete [72]. In the temperature range tested, the fundamental factor governing the residual strength of concrete is the dehydration and rehydration of cement. Changes that occur in aggregates [85], in addition to obvious thermal expansion, minimally influence the above-mentioned strength. In assessing the deterioration of the concrete strength after a fire, an aggregate type is not a deciding factor. However, it should be noted that the aggregate type influences the spalling. The incompatibility of strains between hardened cement paste and aggregates that cause thermal instability depends on the type of aggregate [86]. The initial moisture state is crucial for flint aggregates due to their low porosity, and the build-up of vapor pressure causes explosive spalling in the temperature range of 150 to 450 °C [87].

### 7.2. Cement Dosage and Type

Concrete strength is among other functions a function of the water to cement ratio, so naturally, it influences residual strength. Very few papers tackle cement dosage, and even fewer address cement types. In [46], concrete samples with three different cement dosages and the same w/c ratio were tested. Normal Portland cement with the addition of fly ash was used; it can be classified as CEM II/B-V. Two different cooling regimes were used: inside the furnace and at room temperature. Tests were performed directly after cooling and after 30 days of re-curing. Analyzing the test results, one can conclude that the cement dosage is not influencing the residual strength, as all mixtures behave in a very similar way. In [24], the influence of the w/c ratio on the residual strength was tested. Two types of specimens with different w/c ratios were used (both using CEM I). The tests were performed after 7 days of re-curing. The results show that although the difference between the w/c ratios is substantial (17%), the influence on relative residual strength is negligible. In [50], three different w/c ratios: 0.35, 0.5, and 0.55 were tested. Specimens were heated to 600 °C, cooled down, and tested after various re-curing times. Results for w/c of 0.5 and 0.55 are almost identical, and for 0.35, the initial residual strength is much lower but the increment is similar for all w/c ratios.

Other w/c ratios (0.31 and 0.45) were examined in [42]. For all peak temperatures ranging from 200 to 800 °C, the w/c ratio has been shown to be insignificant when considering its influence on relative residual strength (Table 11). In [88], concrete with a w/c ratio of 0.22, 0.33, and 0.57 at temperatures up to 450 °C was tested. The results showed that the loss of initial strength was lowest for 0.22 (approximately 20%) and higher for 0.33 and 0.57 (approximately 30%). In [82], research on the w/c ratio was performed by exposing three different concrete mixes, with w/c ratios of 0.6, 0.42, and 0.27, to temperatures ranging from 150 to 900 °C. The heating rate was set at 3 °C/min, and the exposure time at the peak temperature was 1 h. The results show that the smaller w/c ratios perform slightly better and maintain more strength. A similar test was performed in [70]; concretes with w/c ratios of 0.5 and 0.7 were exposed to elevated temperatures (ranging from 200 to 1000 °C), and after 28 days of re-curing, residual strength was tested. The results showed that w/c influences residual properties in higher temperature registers, i.e., 600 °C and above. Higher w/c ratios resulted in lower residual strength. Both [89,90] present the influence of the w/c ratio on the residual strength of the concrete after exposure to 500 °C for 1 h and 4 h, respectively. Concrete mixes were prepared with normal and recycled aggregates. The results showed that a lower w/c is beneficial for residual strength, especially for recycled aggregate concrete.

In [59], concretes with different pozzolanic materials used as a partial replacement for Portland cement were tested. These were natural pozzolana and lignite fly ash. The conclusion was made that samples with pozzolanic additives are more sensitive to high temperatures, especially in the temperature magnitude of 200 to 400 °C. For 200 °C, OPC concrete registered a 25% reduction in strength, while in concretes with pozzolanic materials, this reduction ranged from 38 to 50%. Taking into account 400 °C, the disproportion was smaller: 50 to 65%. This behavior can be explained by the higher amount of calcium aluminates hydrate (loses part of its combined water at 105 °C), calcium aluminate sulfate hydrate (dehydrates at 150 °C), and amorphous tobermorite gel (dehydrates at 120 °C) in OPC–pozzolana and OPC–fly ash paste mixtures. The amount of strength gained in the re-curing period is dependent on additives, where OPC concrete regains strength faster than concrete with pozzolanic additives.

Four types of concrete with fly ash replacement for Portland cement were tested in [48]. The influence of the amount of fly ash directly after cooling was tested. Replacement ratios ranged from 10 to 40%. The results showed that there is no correlation between the residual strength directly after cooling and the quantity of fly ash. In [91], the influence of fly ash and metakaolin on the residual strength of HSC was tested. The results reveal that there is no large difference in residual strength directly after cooling for all mixes.

**Figure 15:**
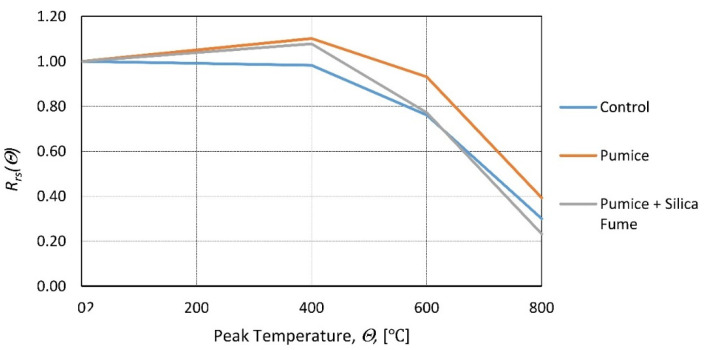
Relative residual compressive strength as a function of the peak temperature for different cement replacements according to [94].

The role of peak temperature and fly ash dosage on the residual strength of lightweight concrete was examined in [92]. The level of importance determined by the Anova method was extremely favorable to the peak temperature, showing that the fly ash quantity impact was minimal. In [93], fly ash dosage did not influence the self-compacting concrete residual strength for peak temperatures up to 300 °C. In [94], research on the influence of finely ground pumice and silica fume on the residual properties of concrete was carried out. Specimens with different dosages of FGP (finely ground pumice) and SF (silica fume) were exposed to high temperatures ranging from 400 to 800 °C and then tested. The results indicate (Figure 15) that FGP additions are beneficial for residual strength, while SF slightly reduces residual strength. A similar conclusion was reached regarding SF in [95].

**Figure 16:**
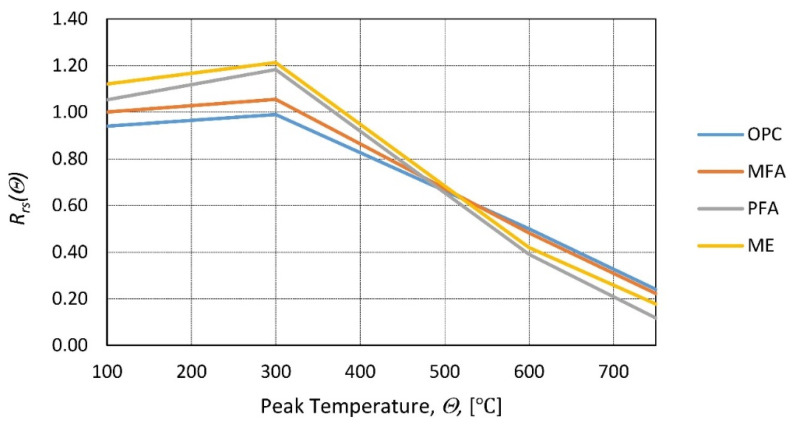
Relative residual compressive strength as a function of the peak temperature for different cement replacements (ME—natural pozzolan, PFA—high calcium fly ash, MFA—low calcium fly ash, OPC—ordinary Portland cement) according to [69].

In [69], the residual strength of the samples with pozzolanic cement replacement was tested. Three types of binder were chosen: the replacement of ME (natural pozzolan), PFA (high calcium fly ash), and MFA (low calcium fly ash), and ordinary Portland cement (OPC) was proposed to be 10 and 30% high. The difference in residual strength induced by the replacement ratio of Portland cement was minimal, so only types of replacement binder were considered. The samples were heated to temperatures of 100 to 750 °C, and the heating rate was set at 2.5 °C/min. After exposure to a peak temperature of 2 h, the samples were naturally cooled inside of the furnace. The residual compressive strength was investigated, and the conclusion was reached that the type of pozzolanic replacement is important only in the lower temperature range (100–400 °C) (Figure 16).

**Figure 17:**
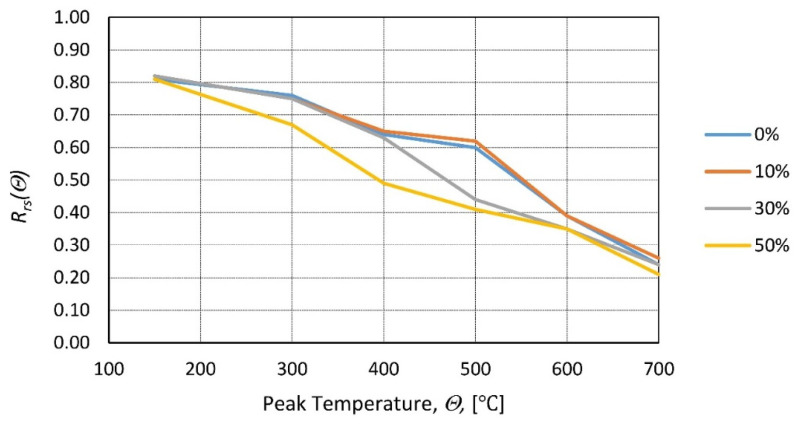
Relative residual compressive strength as a function of the peak temperature for different GGBFS replacement ratios (0, 10%, 30%, and 50%) according to [96].

Another cement replacement, ground granulated blast furnace slag (GGBFS), was investigated in [96]. Mixes were made with the replacement ratios of 10, 30, and 50%. After exposure to elevated temperature (ranging from 150 to 700 °C) and natural cooling in the furnace, compressive tests were performed. The results presented in Figure 17 demonstrate that for low peak temperatures (below 400 °C), replacement of residual strength is insignificant. For higher temperature registers, mixes with the GGBSF replacement resulted in a lower residual strength. The higher replacement ratio resulted in lower mechanical properties.

The available test data prove that the w/c ratio does not have a big influence on the residual strength; only in cases of drastically different ratios, the initial strength differs by a noticeable margin. The rate at which the concrete regains strength is similar for all tested w/c ratios. The cement type (binder additives) is crucial; pozzolans or slag [97] plays an important role in the strength of the cement paste after a fire. More research is needed to fully explain the influence it has on residual strength.

### 7.3. Additives and Fibers

**Figure 18:**
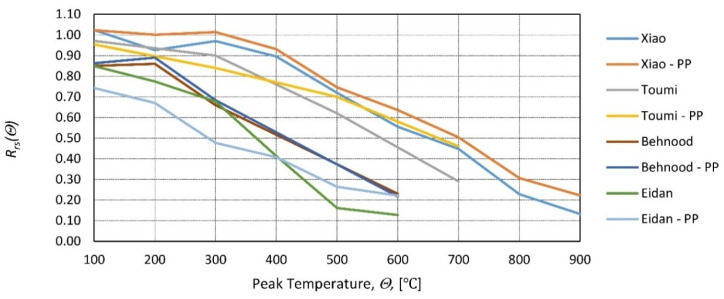
Relative residual compressive strength as a function of peak temperature—with and without PP fibers by [29]—Xiao, [23]—Toumi, [105]—Behnood, and [106]—Eidan.

**Figure 19:**
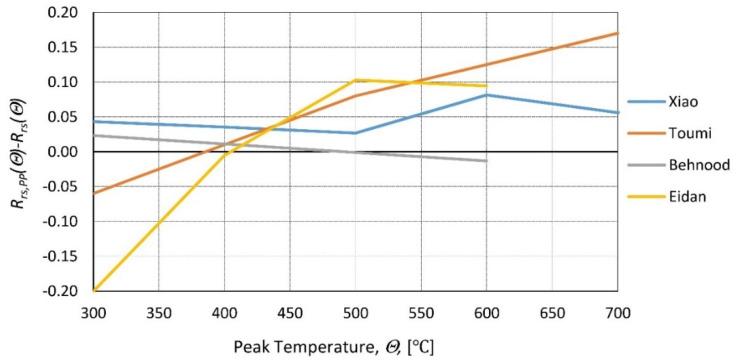
The difference in relative residual compressive strength with and without PP fibers by [29]—Xiao, Toumi [23], Behnood [105], and Eidan [106].

Many additives can improve concrete properties. Macro-additives, such as polypropylene or steel fibers [98] and micro-additives, such as reactive powder [99] or palm oil fuel ash [55], are only a few examples. In [100], the influence of superplasticizer, hardening accelerator, setting retarders, and air entertainers was found to be minimal (only air entertainers showed a noticeable decrease in residual properties). A similar conclusion can be reached by analyzing the paper [101]. Polypropylene (PP) fibers are said to explicitly improve concrete strength at elevated temperatures. PP (polypropylene) fibers melt and create channels that help release the internal water pressure that was built due to the increase in temperature [102]. Without a doubt, it increases the strength of hot concrete, but its influence on residual strength is less pronounced [103,104]. In [23,29], the residual strength of HSC was tested with and without PP fibers. The tests were performed after cooling, and the same conclusion was reached: PP fibers increase residual strength by a small margin (Figure 18 and Figure 19). In [105,106], the influence of different dosages of PP fibers on the residual strength of HSC was tested. The results showed that the differences between various dosages are limited (Figure 18 and Figure 19). The marginal influence of PP and steel fibers on the residual strength of NSC was also reached in [107].

PP fibers improve residual compressive strength directly after cooling but to a very limited degree. It should not be taken into account when assessing the residual strength of concrete. More tests need to be performed on the influence of PP fibers on the re-curing rate. Channels made by melted PP fibers are impossible to repair, as they do not regenerate. The pore structure of concrete without PP can be restored to a value similar to the initial one, while concrete with PP cannot regain its previous state. In [108], it was suggested that microchannels created in place of melted PP fibers have a positive effect on water re-curing of concrete, as they accelerate the water diffusion rate but negatively impact the residual strength of air re-cured concrete. The influence of steel and PP fibers on NSC and residual properties of HSC was studied in [109], and it was found that steel fibers have a minimal effect on NSC and change the spalling temperature to a higher level. The use of PP fibers increased the spalling resistance for all samples, but a negative effect on residual mechanical properties was noticed. In [110] the influence of steel fibers on residual strength at very high temperatures (900–1200 °C) was analyzed, and a minimal influence was observed. Concrete with glass and steel fibers tests were performed in [111]. Up to 30%, higher compressive strength was noted for steel fiber concrete (for glass fiber, up to 20%). This increase was especially visible for the 300–500 °C temperature range. In [112], PP fibers in concrete mixes were tested, and the results showed that thermal behavior and stability are not influenced by type and dosage. Similar research should be conducted on residual mechanical properties.

## 8. Porosity

**Figure 20:**
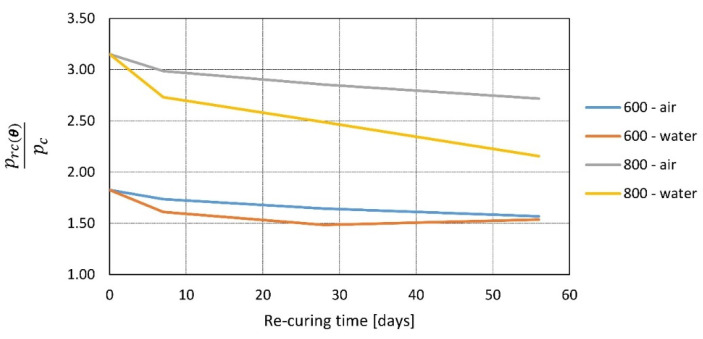
Relative porosity as a function of re-curing time with different re-curing methods (air re-curing and water re-curing) and different peak temperatures *θ* = 600 and 800 °C according to [60].

Porosity, pore size, and pore distribution are the primary factors influencing the strength, durability, and permeability of concrete. Research already done in this area is collected in Table 12. High-temperature exposure increases the porosity and coarsening of the pore structure of concrete [58]. The dehydration process that occurs in the C-S-H (calcium–silicate–hydrate) gel decreases its volume and subsequently increases its porosity. Although up to 200 °C, a slight expansion of the cement paste is observed, above this temperature, rapid shrinkage occurs. This phenomenon greatly influences the evolution of porosity. In [60], porosity was measured using the mercury intrusion porosimeter (MIP). Compared to the preheating values, porosity directly after cooling was two times higher for 600 °C and three times higher for 800 °C. However, post-fire re-curing significantly reduced the porosity by the rehydration of particles that filled capillaries. Lower porosity results in a dense microstructure and better mechanical properties. When comparing the results presented in [60] (Figure 20), lower initial porosity leads to slightly higher relative residual strength. If two concretes with different initial porosities are compared with relation not to initial but after cooling strength and porosity, then re-curing gains are very similar (Table 13).

In [27], an MIT (mercury intrusion porosimetry test) test was used to measure changes in connected porosity (open pore network). Tests were performed after cooling. The results compared to previous research are consistent as presented in Table 14. The relative porosity increases at a rate similar to that observed in [60], and the relative residual strength at the corresponding temperature is similar. 

**Figure 21:**
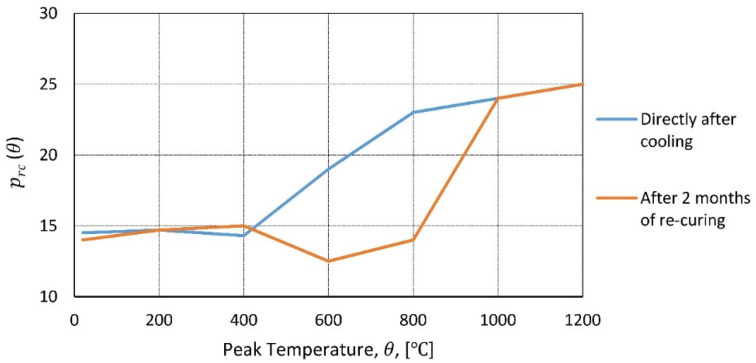
Total porosity as a function of the peak temperature and curing according to [113].

An interesting relation was observed in [113], comparing porosity at relative peak temperature, between tests performed directly after cooling (a) and 2 months of water curing (b). Porosity in the temperature range of up to 400 °C is constant. In the range from 400 to 800 °C, porosity is increasing for the sample that was tested directly after cooling, but the porosity level of the water-cured sample is still constant. From 800 to 1000 °C, (a) is slowly increasing, while (b) noted rapid growth, and at temperatures above 1000 °C, the type of curing is irrelevant (Figure 21).

Porosity is rapidly growing, with temperature increasing above 400 °C and having a great impact on the strength of concrete. The calculated correlation factors between porosity after exposure and residual strength shows a significant negative porosity influence (Table 15). This relationship can be nonlinear, as the Pearson and Spearman coefficients are not very high. It was noted that the porosity growth is higher for HSC [114]. Nevertheless, damages caused by dehydration can be repaired by rehydration of the concrete. With an appropriate curing method applied to concrete exposed to peak temperatures not exceeding 800 °C, porosity can be regenerated to levels before heating. Taking these facts into account, the initial porosity of concrete plays a marginal role in the relative residual strength. The relative growth of residual strength (with relation to strength directly after cooling) of concretes with different initial porosities is very similar.

## 9. Age of Concrete at Exposure

Fire can happen in a building regardless of its age. Both very old buildings and new buildings (or even still under construction) can experience exposure to fire situations. The behavior of concrete at high temperatures will be different in the mentioned cases. Moisture and the amount of concrete that was already hydrated influence both hot and residual strength but also the re-curing rate. In [115,116], concrete that was exposed to an elevated temperature at an early age (from 1 to 28 days after casting) was tested. Residual strength tests showed that young concrete had a better recovery rate (with the exception of 1-day-old concrete, as it did not have enough strength to withstand high temperature, and damage during the heating period was considerable. In [117], the strain-hardening cementitious composite was tested, and a similar conclusion was reached.

## 10. Load Level at Exposure

The level of load at exposure is an important issue that needs to be addressed, as every building is constantly subjected to loads. Strength at high temperature is positively affected by load level, as it reduces the speed of decrease of strength [118,119,120]. In [121], the influence of preload on residual strength was analyzed. There were three preload levels (0, 20, and 40% of the ultimate load at room temperature), and after exposure to high temperature and cooling, compressive tests were performed. The results showed that preload results in a higher residual strength (for 20% of preload increase, it is approximately 15%). This can be attributed to the restriction of thermal expansion by acting on the load, thus minimizing concrete damage. In [122], free expansion deformation of unstressed specimens exposed to high temperature at different heating rates was investigated. The linear expansion rate (LER) measured at high temperature was a linear function of temperature and did not depend on the heating rate. Restraining expansion and thus minimalizing internal cracking can greatly benefit residual strength. Thus, compressive stress plays a positive role.

## 11. Heat Accumulation Factor

The hot and residual strengths of concrete depend on the dehydration of the cement gel. Dehydration is mainly related to high exposure to heat, both the peak temperature and the exposure time. The factor that evaluates high-temperature exposure is the heat accumulation factor. It is defined as the area under the temperature–time curve. This idea was proposed in [46], and various studies [60,123,124,125] show that cement paste decomposition begins when the temperature exceeds 400 °C. Thus, the heat accumulation factor influencing the strength of the concrete should take into account only temperatures above 400 °C. Exposure to 200 °C will damage the concrete to an incomparably smaller degree than short exposure to 400 °C.

**Figure 22:**
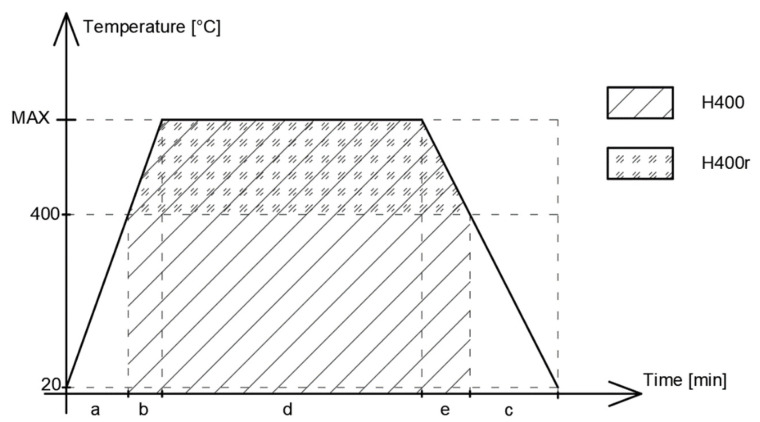
Diagram of the calculation of the heat accumulation factor, *H*^400^ and *H*^400*r*^.

The heat accumulation factor can be calculated in two ways (Figure 22), as originally proposed in [46] by the use of Equation (1) $\left(H^{400}\right)$ and by the method proposed by the authors $\left(H^{400r}\right)$:

(1) $$H^{400}=\int_{tr}^{ts}T\left(t\right)dt$$

(2) $$H^{400r}=\int_{tr}^{ts}\left(T\left(t\right)-400\right)\;dt.$$

**Figure 23:**
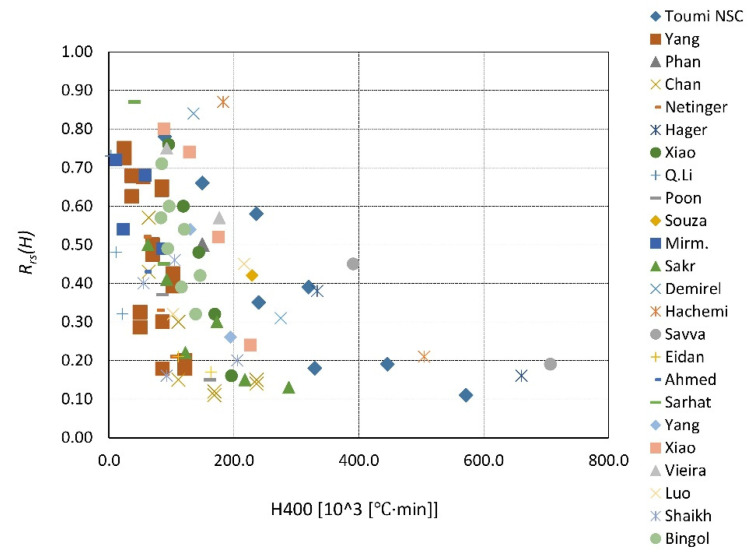
*H*^400^ coefficient for NSC by different authors.

**Figure 24:**
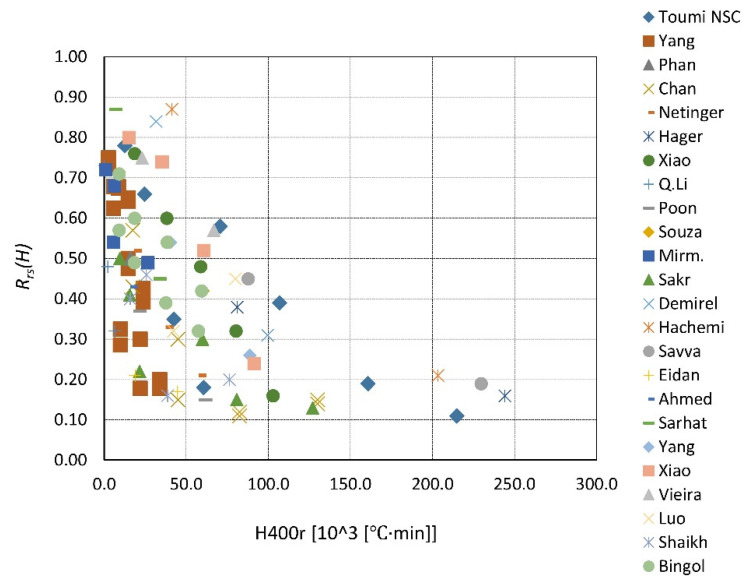
*H*^400*r*^ coefficient for NSC by different authors.

**Figure 25:**
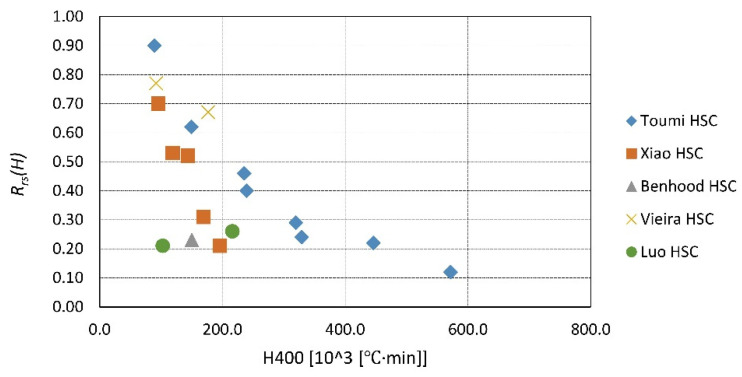
*H*^400^ coefficient for HSC by different authors.

**Figure 26:**
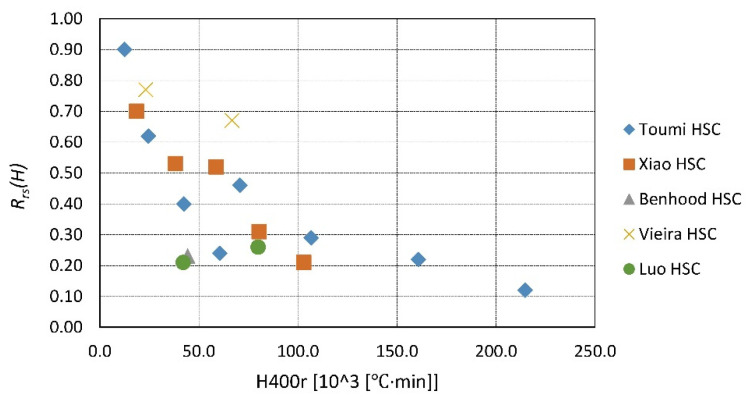
*H*^400*r*^ coefficient for HSC by different authors.

**Figure 27:**
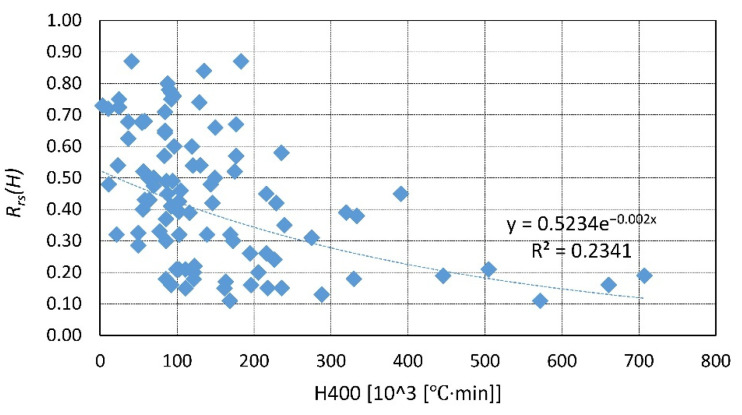
*H*^400^ coefficient for NSC—summary and fitting.

**Figure 28:**
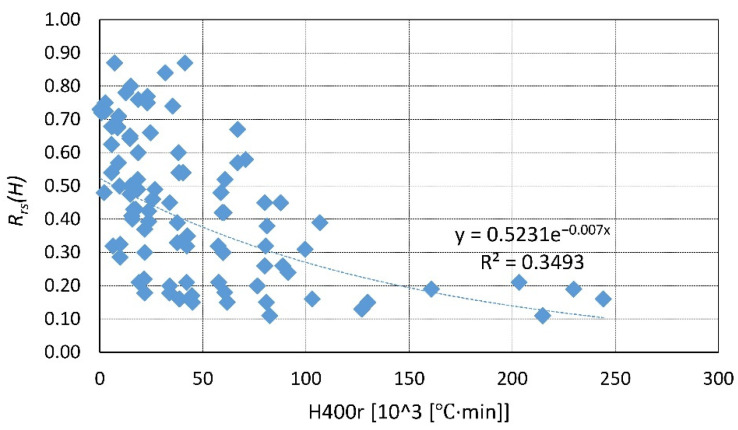
*H*^400*r*^ coefficient for NSC—summary and fitting.

**Figure 29:**
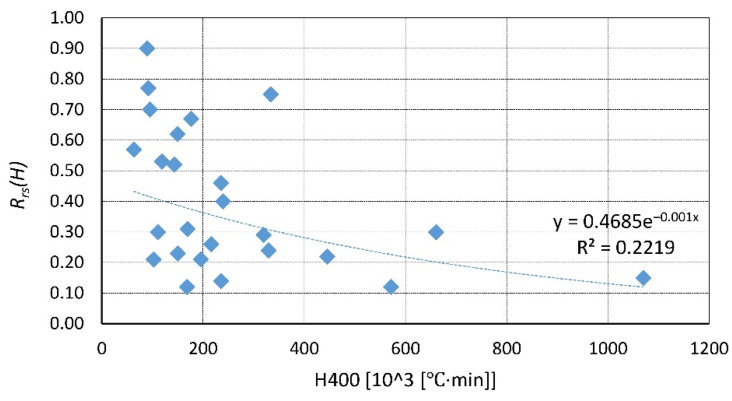
*H*^400^ coefficient for HSC—summary and fitting.

**Figure 30:**
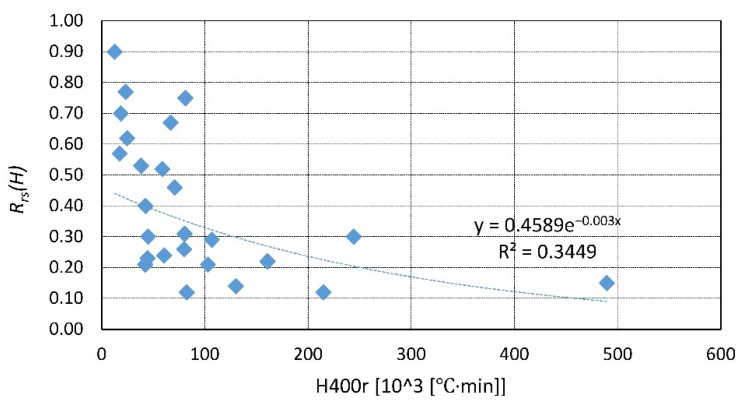
*H*^400*r*^ coefficient for HSC—summary and fitting.

A summary of the existing data is presented in Figure 23, Figure 24, Figure 25, Figure 26, Figure 27, Figure 28, Figure 29 and Figure 30. Heating and cooling rates were treated as constant to simplify the calculation. It is visible that *H*^400^ follows a certain decreasing line (Figure 23 and Figure 25), which was easy to predict, but the scatter is big, and there is no easy way to generalize the results. *The H*^400*r*^ is less scattered, but it is still hard to derive a solid function (Figure 24 and Figure 26). A comparison between *H*^400^ and *H*^400*r*^ is presented in Figure 27 and Figure 28 for NSC and Figure 29 and Figure 30 for HSC. The three correlation factors presented in Table 16 demonstrate the superiority of the modified heat accumulation factor (Equation (2)). The values of the factors suggest a nonlinear relationship.

Thus, nonlinear exponential fitting was made. The R^2^ factor (not deciding for nonlinear regression) was again higher in the *H*^400*r*^ variation. However, the data of NSC and HSC behave similarly. With this in mind, for future reference, NSC and HSC can be treated identically, and separation is unnecessary.

## 12. Discussion

Although experimental research on the residual strength of concrete is extensive, the results appear to be incomplete. The main factors influencing residual strength (peak temperature, heating time, cooling regime, post-heating re-curing, load level at exposure) are already identified. Other factors, but with less pronounced effects (heating rate, type of aggregate, cement type, and dosage) have also been examined. However, some factors do not contribute to the strength, such as common additives, or the influence is not straightforward, such as porosity. However, with this in mind, experimental research can be limited to the temperature range 300–700 °C because the residual strength for temperatures up to 300 °C and more than 750 °C can be obtained only on the basis of the peak temperature. Moreover, the limit of two hours of high-temperature exposure can be used, as the main strength loss occurs within this period, and later, the impact is minimal.

Both essential factors influencing the residual strength (i.e., peak temperature and heating time) exhibit a negative linear influence on the residual strength, while for the other factors, the influence is not so straightforward, as the correlation factors are smaller. Thus, nonlinear functions would properly govern these relationships. Furthermore, the influence of the peak temperature on the residual strength is not as direct as on the hot concrete strength. Only the strength directly after cooling is highly dependent on the peak temperature reached. A similar conclusion is drawn for the cooling regime; e.g., for water cooling, its negative impact is apparent in the early stage of recurring but for the longer re-curing time, the impact diminishes. The influence of rapid cooling is also limited to the external layers, and it is not deciding for whole structure load capacity.

What should be stressed is the ability of concrete to regain its strength due to the rehydration of cement paste. Therefore, re-curing time and type are crucial for assessing the residual strength of concrete. From this perspective, it is not surprising that the influence of the w/c ratio can be omitted. However, there are reports where a lower w/c ratio results in a higher residual strength. This problem may be posed by cement type, as there is not enough comparative research focusing on the type of cement and its influence on residual strength. There exists also an indirect relationship between rehydration and porosity that requires further research. Porosity is notably higher after high-temperature exposure, and reversal is possible if the temperature was not greater than 800 °C. Moreover, lower porosity results in higher residual strength directly after cooling, but after re-curing, this difference is equalized.

The time after cooling and its type also regulate the residual strength. Directly after water cooling, a lower residual strength is obtained. Nevertheless, the re-curing significantly reduces the influence of cooling type. However, additional consideration of these phenomena is necessary for the external part of an element that is severely damaged. The internal part, which is crucial from a residual strength point of view, is immune to damages caused by different types of cooling. The interaction between layers of heated and cooled concrete should also be studied. The transient temperature field and associated strain and stress can contribute to material damage and strength reduction. Moreover, the possible volume changes due to chemical reactions and thermal expansion would also be considered.

There is also a need for more research on the influence of PP fibers on regaining residual strength. Changes in concrete structure left by PP fibers are evident, and their impact needs to be assessed. More research is also needed to determine the relation between preload level and residual strength, as it can be important in practice.

Since the ability of concrete to regain its strength due to rehydration determines the residual strength, only temperatures above 400 °C should be taken into account, because the decomposition of cement paste starts at this temperature. The modified heat accumulation factor *H*^400*r*^ gives more coherent results than the unmodified *H*^400^ as demonstrated by correlation factors, and therefore, it can be used to assess the residual strength of concrete.

## 13. Conclusions

The research presented in the paper signifies the determination of the peak temperature and the heating time of the residual strength. The other factors do not directly influence the residual strength. However, as concrete regains its strength due to the rehydration of cement paste, the re-curing time and type are also crucial factors.

The proposed modified heat accumulation factor can be considered as a measure that collects all influences. However, more research needs to be done to increase accuracy and prove that the modified accumulation factor is universal, i.e., if in changing experimental conditions, the same values of the factor are associated with the same values of the residual strength. If not, other measures of damage should be considered.

If the other measures of damage are proposed, they should take into account that the derivative of residual strength regarding heating time is constant, and the residual strength can be described as a logarithmical function of re-curing time and type. Thus, to create a function that will precisely assess the residual strength of concrete, one needs to solve an equation system that takes all influential variables into account. Considering only part of the variables will result in an approximate solution. A comprehensive function of residual strength can be found only after the above-mentioned factors, extended by necessary research, are rationalized by mathematical function, compressed to the equation system, and then solved.

## Figures and Tables

**Table 1:** Summary of research on the peak temperature.

Author	Citation	Sample Type	Sample Size	Concrete Strength	Temp. Range	Heating Time	Heating Rate	Age of Concrete at Exposure
Toumi	24	Cubic	100 mm	NSC, HSC	300–700 °C	3–9 h	10 °C/min	28 days
Yang	25	Cylindrical	D 100 mm H 200 mm	NSC	400–600 °C	0–2 h	2.5 °C/min	90 days
Phan	26	Cylindrical	D 100 mm H 200 mm	NSC, HSC	100–450 °C	5 h 30 min	5 °C/min	200 + days
Netinger	27	Beam	40 mm × 40 mm × 160 mm	NSC	200–1000 °C	1 h 30 min	-	28 days
Hager	28	Cubic Cylindrical	Cubic: 150 mm Cyl.: D 100 mm H 200 mm	HSC	200–1000 °C	3 h	0.5 °C/min	90 days
Krzemień	29	Cubic	150 mm	NSC	200–1000 °C	3 h	0.5 °C/min	120 days
Xiao	30	Cubic	100 mm	HSC	100–900 °C	3 h	ISO-834	NA
Chan	31	Cubic	100 mm	NSC, HSC	400–1200 °C	1 h	BS476:Part20:1987	90 days
Tolentino	32	Cylindrical	D 100 mm H 200 mm	NSC, HSC	600 °C	2 h	0.5 °C/min	NA
Xiao	34	Cubic	100 mm	HSC	200–800 °C	2–3 h	25 °C/min	150 days

**Table 2:** Correlation factors between peak temperature and relative residual strength for two different heating times at peak temperature as presented by various authors.

Heating Time	1 h	3 h
Pearson	−0.90	−0.95
Spearman	−0.94	−0.96
Kendall	−0.82	−0.87

**Table 3:** Summary of the research for heating time.

Author	Citation	Sample Type	Sample Size	Concrete Strength	Temp. Range	Heating Time	Heating Rate	Age of Concrete at Exposure
Toumi	24	Cubic	100 mm	NSC, HSC	300–700 °C	3–9 h	10 °C/min	28 days
Yang	25	Cylindrical	D 100 mm H 200 mm	NSC	400–600 °C	0–2 h	2.5 °C/min	90 days
Pertiwi	38	Cubic	150 mm	NSC	400–600 °C	3–9 h	NA	28 days
Wu	39	Cylindrical	D 100 mm H 200 mm	NSC	100–600 °C	1–6 h	5 °C/min	28 days
Mohamedbhai	40	Cubic	100 mm	NSC	200–800 °C	1–4 h	-	84 days

**Table 4:** Correlation factors between the time maintained at the peak temperature and the relative residual strength for a peak temperature of 500 °C, presented by various authors.

Correlation Coefficient	Coefficient Value
Pearson	−0.98
Spearman	−0.86
Kendall	−0.75

**Table 5:** Correlation factors between the heating rate and the relative residual strength for the peak temperature of 500 °C, presented by various authors.

Correlation Coefficient	Coefficient Value
Pearson	0.21
Spearman	0.21
Kendall	0.17

**Table 6:** Summary of research on the cooling regime.

Author	Citation	Sample Type	Sample Size	Concrete Strength	Temp. Range	Heating Time	Cooling Regime	Age of Concrete at Exposure
Kowalski	42	Cylindrical	D 100 mm, H 200 mm	NSC	330–550 °C	3–5 h	Air cooling Water cooling	130 days
Peng	43	Cubic	100 mm	HSC	200–800 °C	1 h	Air cooling Water cooling	56 days
Yang	44	Cubic	100 mm	NSC	200–800 °C	3 h	Air cooling Water cooling	90 days 120 days
Husem	45	Beam	40 mm × 40 mm × 160 mm	NSC, HSC	200–1000 °C	1 h	Air cooling Water cooling	28 days
Mendes	46	Cylindrical	D 100 mm, H 200 mm	NSC	400–800 °C	1 h	Air cooling Water cooling	90 days
Bingol	47	Cylindrical	D 100 mm, H 200 mm	NSC	100–700 °C	3 h	Air cooling Water cooling	28 days
Li	48	Cubic	100 mm	HSC	100–800 °C	0	Air cooling	90 days
Luo	49	Cubic	100 mm	NSC, HSC	800–1100 °C	1 h	Air cooling Water cooling	90 days
Shaikh	50	Cylindrical	D 100 mm, H 200 mm	NSC	200–800 °C	2 h	Air cooling Water cooling	56 days
Wang	51	Cubic	100 mm	NSC	200–800 °C	3 h	Air cooling Water cooling	130–142 days
Li	52	Cylindrical	D 100 mm, H 200 mm	NSC	150–750 °C	2 h 30 min	Air cooling Water cooling	90 days
Karakoç	53	Cylindrical	D 100 mm, H 200 mm	NSC	700 °C	1 h	Air cooling Water cooling	1 year

**Table 7:** Comparison of the relative residual strength for different cooling methods according to [46].

	Re-Curing Time	0 Days	30 Days
Cooling Method			
$\frac{f_{rc,AC}}{f_{c,20}}$		0.77	0.82
$\frac{f_{rc,FC}}{f_{c,20}}$		0.65	0.87

**Table 8:** Difference between the relative residual strength for air and water cooling according to [49,50].

			$\frac{\left(f_{rc,AC}-f_{rc,WC}\right)}{f_{c,20}}\cdot 100\%$							
		Temp.	150 °C	200 °C	300 °C	400 °C	450 °C	600 °C	750 °C	800 °C
	Re-Curing									
[50]	0 days		1.5%	-	2.5%	-	5.8%	14.0%	13.6%	-
	30 days		0.0%	-	−0.2%	-	2.9%	4.5%	1.5%	-
	60 days		0.1%	-	2.9%	-	3.7%	−6.3%	−3.0%	-
	90 days		−1.1%	-	0.5%	-	1.7%	−6.5%	−2.5%	-
[49]	30 days		-	5.5%	-	6.7%	-	-	-	−1.5%

**Table 9:** Summary of research on the post-fire re-curing.

Author	Cit.	Sample Type	Sample Size	Concrete Strength	Temp. Range	Heating Time	Cooling Regime	Re-Curing Regime	Re-Curing Time	Age of Concrete at Exposure
Li	52	Cylindrical	D 100 mm, H 200 mm	NSC	150–750 °C	2 h 30 min	Air cooling Water cooling	Air re-curing	30–90 days	90 days
Papayianni	61	Cylindrical	D 150 mm, H 300 mm	NSC	200–800 °C	3 h	Air cooling	NA	1–90 days	180 days
Poon	62	Cubic	100 mm	NSC, HSC	600–800 °C	1 h	Air cooling	Air re-curing Water re-curing	7–56 days	60 days
Souza	63	Cylindrical	D 100 mm, H 200 mm	NSC	300–600 °C	2 h 2 h 40 min	Air cooling	Air re-curing Water re-curing	28–112 days	100 days
Lin	64	NA	NA	NSC	400–1000 °C	2 h	NA	Air re-curing Water re-curing	7–180 days	90 days
Mirmomeni	65	Cylindrical	D 40 mm, H 40 mm	NSC	300–600 °C	15 min 2 h	Air cooling Water cooling	Water re-curing	2–28 days	28 days
Horiguchi	66	NA	NA	HSC	200–400 °C	2 h	NA	Air re-curing Water re-curing	90–180 days	NA
Park	67	Cylindrical	D 100 mm, H 25 mm	NSC	300–700 °C	1 h	Water cooling	Air re-curing Water re-curing	7–30 days	28 days

**Table 10:** Correlation factors between re-curing time and the relative residual strength for the peak temperature of 500 °C, presented by [61,63].

Correlation Coefficient	Coefficient Value
Pearson	0.617
Spearman	0.777
Kendall	0.661

**Table 11:** Relative residual compressive strength for different w/c ratios (0.58 and 0.68) and the difference between relative residual strengths of different w/c ratios according to [42].

*R*_*rs*_ (*θ*)	w/c = 0.58	Peak temperature			
	Time * [h]	400 °C	500 °C	550 °C	600 °C
	0	0.93	0.73	0.63	0.33
	1	0.83	0.68	0.48	0.30
	2	0.70	0.65	0.43	0.20
	w/c = 0.68	Peak temperature			
	Time * [h]	400 °C	500 °C	550 °C	600 °C
	0	0.86	0.75	0.68	0.29
	1	0.82	0.68	0.50	0.18
	2	0.71	0.64	0.39	0.18
$f_{rc,20}\left(\frac{w}{c}=0.58\right)-f_{rc,20}\left(\frac{w}{c}=0.68\right)$	Difference	Peak temperature			
	Time * [h]	400 °C	500 °C	550 °C	600 °C
	0	6.79%	−2.50%	−5.36%	3.93%
	1	0.36%	−0.36%	−2.50%	12.14%
	2	−1.43%	0.71%	3.21%	2.14%

* Time maintained at peak temperature.

**Table 12:** Summary of the research on porosity.

Author	Citation	Sample Type	Sample Size	Concrete Strength	Temp. Range	Heating Time	Porosity	Age of Concrete at Exposure
Hager	28	Cubic Cylindrical	Cubic: 150 mm Cyl.: D 100 mm, H 200 mm	HSC	200–1000 °C	3 h	1.4–2%	90 days
Poon	62	Cubic	100 mm	NSC, HSC	600–800 °C	1 h	6.69–9.52%	60 days
Chromá	115	Beam	40 mm × 40 mm × 160 mm	NSC	200–1200 °C	2 h	15%	28 days
Chan	116	Cubic	NA	NSC, HSC	800–1100 °C	1 h	NA	90 days

**Table 13:** Porosity and relative porosity as a function of re-curing time for different concrete mixes (CC—control sample, FA30—30% of cement replaced by fly ash) according to [49].

		Directly after Cooling—t = 0 Days				Re-Curing—Air—t = 28 Days				Re-Curing—Water—t = 56 Days			
	Peak Temp. [°C]	θ = 600	θ = 600	θ = 800	θ = 800	θ = 600	θ = 600	θ = 800	θ = 800	θ = 600	θ = 600	θ = 800	θ = 800
	Initial Porosity [%]	$p_{rc}\left(\theta ,0\right)$	*R*_*rs*_ (*θ*,*t*)	$p_{rc}\left(\theta ,0\right)$	*R*_*rs*_ (*θ*,*t*)	$p_{rc}\left(\theta ,t\right)$	*R*_*rs*_ (*θ*,*t*)	$p_{rc}\left(\theta ,t\right)$	*R*_*rs*_ (*θ*,*t*)	$p_{rc}\left(\theta ,t\right)$	*R*_*rs*_ (*θ*,*t*)	$p_{rc}\left(\theta ,t\right)$	*R*_*rs*_ (*θ*,*t*)
HS-CC	9.52	18.3	0.58	17.71	0.24	16.56	0.67	26.66	0.37	16.96	0.69	23.04	0.52
HS-FA30	6.69	11.3	0.67	10.44	0.32	10.1	0.77	19.6	0.47	7.96	0.93	11.91	0.79
		$\frac{p_{rc}\left(\theta ,t\right)}{p_{rc}\left(\theta ,0\right)}$	$\frac{Rrs\;\left(\theta ,t\right)}{Rrs\;\left(\theta ,0\right)}$	$\frac{p_{rc}\left(\theta ,t\right)}{p_{rc}\left(\theta ,0\right)}$	$\frac{Rrs\;\left(\theta ,t\right)}{Rrs\;\left(\theta ,0\right)}$	$\frac{p_{rc}\left(\theta ,t\right)}{p_{rc}\left(\theta ,0\right)}$	$\frac{Rrs\;\left(\theta ,t\right)}{Rrs\;\left(\theta ,0\right)}$	$\frac{p_{rc}\left(\theta ,t\right)}{p_{rc}\left(\theta ,0\right)}$	$\frac{Rrs\;\left(\theta ,t\right)}{Rrs\;\left(\theta ,0\right)}$	$\frac{p_{rc}\left(\theta ,t\right)}{p_{rc}\left(\theta ,0\right)}$	$\frac{Rrs\;\left(\theta ,t\right)}{Rrs\;\left(\theta ,0\right)}$	$\frac{p_{rc}\left(\theta ,t\right)}{p_{rc}\left(\theta ,0\right)}$	$\frac{Rrs\;\left(\theta ,t\right)}{Rrs\;\left(\theta ,0\right)}$
HS-CC		1.00	1.00	1.00	1.00	0.90	1.16	1.51	1.54	0.93	1.19	1.30	2.17
HS-FA30		1.00	1.00	1.00	1.00	0.90	1.15	1.88	1.47	0.71	1.39	1.14	2.47

**Table 14:** Relative residual compressive strength and relative porosity as a function of the peak temperature according to [27].

	Temperature [°C]				
	20	200	400	600	800
$\frac{p_{rc}\left(\theta \right)}{p_{c}}$	1.00	1.65	1.85	2.46	2.74
*R*_*rs*_ (*θ*)	1.00	1.00	0.90	0.70	0.25

**Table 15:** Correlation factors between porosity after exposure and relative residual strength, based on data presented by various authors.

Correlation Coefficient	Coefficient Value
Pearson	−0.697
Spearman	−0.755
Kendall	−0.593

**Table 16:** Correlation factors between heat accumulation factors and relative residual strength based on data presented by various authors.

Coefficient	NSC		HSC	
	*H* ^400^	*H* ^400*r*^	*H* ^400^	*H* ^400*r*^
Pearson	−0.455	−0.543	−0.434	−0.498
Spearman	−0.494	−0.615	−0.500	−0.675
Kendall	−0.335	−0.435	−0.363	−0.490

## Supplementary Materials

The following are available online at https://www.mdpi.com/article/10.3390/ma14164719/s1, Supplementary file.xlsx.

## Abbreviation

Upper case letters	
C-S-H gel	calcium-silicate-hydrate gel
FGP	finely ground pumice
GGBFS	ground granulated blast furnace slag
HSC	high strength concrete
ME	natural pozzolana of volcanic origin, Milos Earth
MFA	Megalopolis fly ash
MIT	mercury intrusion porosimetry test
NSC	normal strength concrete
OPC	ordinary Portland cement
PFA	Ptolemaida fly ash
PP fibers	polypropylene fibers
RCA	recycled concrete aggregate
RCCA	recycled ceramic coarse aggregate
SF	silica fume
Lower case letters	
$f_{rc,T_{t}}\left(\theta \right)$	residual strength of concrete after exposure to elevated temperature (θ) then cooled down and tested in temperature ($T_{t}$)
$f_{c,T_{t}}$	strength of unexposed concrete tested in temperature ($T_{t}$)
$f_{rc,AC}$	residual strength of concrete after exposure to elevated temperature then cooled down to ambient temperature in air and tested
$f_{rc,WC\;}$	residual strength of concrete after exposure to elevated temperature then cooled down in the water to ambient temperature and tested
$f_{rc,WC}\left(t\right)$	residual strength of concrete after exposure to elevated temperature then cooled down in the water for time (t), then cooled down to ambient temperature in air and tested
$f_{rc,FC}$	residual strength of concrete after exposure to elevated temperature then cooled down in closed furnace to ambient temperature and tested
$p_{c}$	porosity of unexposed concrete
$p_{rc}\left(\theta ,\;t\right)$	porosity of concrete after exposure to elevated temperature (θ) then cooled down and tested at a temperature of 20 °C after (t) days of re-curing
$\frac{p_{rc}\left(\theta \right)}{p_{c}}$	relative porosity of concrete tested in 20 °C
w/c	water to cement ratio
Subscripts	
AC	value pertaining to air re-curing
c	value pertaining to unexposed concrete
FC	value pertaining to cooling in closed furnance
rc	residual value
T_t_	temperature of sample in test
WC	value pertaining to water re-curing
